# Impact of image preprocessing on dermatological OCTA vessel segmentation: a DERMA-OCTA study

**DOI:** 10.1117/1.JBO.30.11.116005

**Published:** 2025-11-24

**Authors:** Giulia Rotunno, Massimo Salvi, Julia Deinsberger, Lisa Krainz, Lukasz Bugyi, Benedikt Weber, Christoph Sinz, Harald Kittler, Leopold Schmetterer, Wolfgang Drexler, Mengyang Liu, Kristen M. Meiburger

**Affiliations:** a Politecnico di Torino, Department of Electronics and Telecommunications, Polito BIOMed Lab, Torino, Italy; b Medical University of Vienna, Department of Dermatology, Vienna, Austria; c Medical University of Vienna, Center for Medical Physics and Biomedical Engineering, Vienna, Austria; d The University of Sydney, Melanoma Institute Australia, Sydney, New South Wales, Australia; e SERI-NTU Advanced Ocular Engineering (STANCE) Program, Singapore; f Singapore Eye Research Institute, Singapore; g Academic Clinical Program, Duke-NUS Medical School, Singapore; h Nanyang Technological University, School of Chemistry, Chemical Engineering and Biotechnology, Singapore; i Medical University of Vienna, Department of Clinical Pharmacology, Vienna, Austria; j Fondation Ophtalmologique Adolphe De Rothschild, Paris, France; k Aier Hospital Group, Changsha, China

**Keywords:** skin imaging, optical coherence tomography angiography, segmentation, deep learning, vessel analysis

## Abstract

**Significance:**

Optical coherence tomography angiography (OCTA) offers dye-free, three-dimensional views of skin microvasculature, yet progress in developing reliable and quantitative solutions for vessel architecture analysis is slowed by heterogeneous preprocessing practices, scarce annotated data, and limited evaluation metrics.

**Aim:**

We assess how typical OCTA preprocessing steps influence the accuracy of deep learning vessel segmentation, and we identify network designs and metrics best suited to OCTA dermatological data.

**Approach:**

Experiments use the open DERMA-OCTA dataset containing 330 volumes from different skin conditions; each volume is additionally provided in five progressively pre-processed versions: original, Bscan normalization, projection artifact attenuation, contrast enhancement, and vesselness filtering. Segmentation is performed with representative 2D and 3D deep learning approaches. Besides standard segmentation metrics, evaluation includes the connectivity–area–length index, which proved particularly effective for assessing dermatological vessel segmentation.

**Results:**

The analysis shows that Bscan normalization, projection artifact attenuation, and contrast enhancement incrementally improve segmentation accuracy, whereas vesselness enhancement can impair segmentation performance. Among the tested architectures, 2D models achieved the highest segmentation performance, although 3D approaches proved more effective for deeper tissue layers. Testing across different pathologies revealed challenges in model generalization to varied vascular patterns.

**Conclusions:**

Combining 2D and 3D models and using topology-aware indices provide a full, clinically relevant evaluation of algorithm performance.

## 1 Introduction

Optical coherence tomography (OCT) is a low-coherence interferometric imaging technique that produces microscale cross-sectional images of tissue by detecting back-scattered near-infrared light.^1^ OCT is established in ophthalmology and cardiology, and its role in dermatology is growing, with high-definition and line-field confocal variants enabling noninvasive visualization of epidermal thickness, dermal–epidermal junction morphology, and tumor margins without the need for excisional biopsy.^2^

Optical coherence tomography angiography (OCTA) extends conventional OCT by retrieving motion-related signals from consecutive scans, turning a purely structural technique into one that visualizes and quantifies microvasculature in three dimensions noninvasively and without dyes.^3^ OCTA can indeed provide a high-resolution view of retinal capillary plexuses, making it a useful research and diagnostic tool in ophthalmology, where it has gained insight into diabetic retinopathy, age-related macular degeneration, glaucoma, and venous occlusions.^4^

Recently, researchers have begun applying OCTA to skin for dermatological applications, a much more optically complex tissue than the eye. Multiple scattering layers, heterogeneous refractive indices, and motion can create artifacts that complicate imaging and interpretation. Still, OCTA has already revealed disease-specific microvascular signatures in melanoma,^5^ basal-cell^6^^,^^7^ and squamous-cell carcinoma,^8^^,^^9^ psoriasis,^8^^,^^9^ venous-ulcer margins,^9^ benign melanocytic lesions,^5^^,^^7^^,^^8^ port-wine birthmarks,^10^ wounds,^11^ scars,^7^^,^^12^ burns,^13^^,^^14^ and diabetic foot diseases.^15^^,^^16^ Quantitative biomarkers such as density, diameter, and length are clinically relevant but depend on accurate vessel segmentation. In chronic venous disease (CVD), for instance, OCTA detected: (a) larger vessel radii in early telangiectasia and corona phlebectatica, (b) a progressive fall in vessel length from early disease stage to the ulcer-scar stage, (c) increasing tortuosity beyond skin changes, and (d) marked capillary densification in active ulcers; all observations (a) to (d) pertain to CVD and are reported in Rotunno et al.^17^

Analyzing skin OCTA volumes is technically demanding for several reasons. First, signal quality is highly sensitive to micro-motion and instrument noise, leading to motion artifacts and speckle. Second, skin introduces depth-dependent attenuation and shadowing (e.g., from hair) and strong epidermal backscatter; moreover, OCTA data are always affected by projection artifacts that create false deeper-layer vessel signals arising when fluctuations from superficial blood flow are optically projected onto underlying structures, creating replicas that confound layer-specific analyses. Third, anatomy and acquisition vary widely across body sites, disease phenotypes, and manufacturers (scan protocol, field of view, resolution), causing domain shift and hindering generalization. These factors motivate learning-based approaches that might model context and tolerate artifact variability specifically for cutaneous OCTA volumes. To date, deep learning approaches imported from ophthalmology applications have improved automation, but their performance still hinges on consistent, artifact-reduced input and on large, expertly annotated datasets.^18^^–^^20^ Moreover, OCTA produces 3D volumes, yet most studies still segment 2D slices because artifacts and the near-absence of 3D ground-truth labels in dermatology hamper full volumetric analysis.^19^ In ophthalmology, denoising and threshold-based methods remain standard, whereas newer AI approaches such as CGNet, transformer models, and pseudo-3D pipelines (e.g., 2D Unet reconstructions, layer-attention or projection networks) are emerging.^21^^–^^24^ Dermatological OCTA currently lacks both standardized pre-processing pipelines and publicly available ground-truth data, limiting reproducibility and algorithm training.

To overcome these barriers, DERMA-OCTA, the first open dataset dedicated to cutaneous OCTA, was created and recently published and is freely downloadable.^25^ It comprises 330 volumes from 74 participants imaged at the Medical University of Vienna: 65 patients spanning all Clinical-Etiological-Anatomical-Pathophysiological (CEAP) stages of CVD, 13 healthy controls, and nine individuals with various malignant or benign lesions. Each original 3D scan is accompanied by several processed versions in 2D and 3D, together with expert vessel annotations, creating an unprecedented resource for developing and benchmarking automated skin-vessel analysis.

Using this dataset, we investigate how different data preparation techniques affect vessel segmentation accuracy in both 3D volumes and their corresponding 2D projections. Through a comprehensive set of experiments on DERMA-OCTA, in this work, we aim to address the following research questions:

- How do different preprocessing methods impact deep learning vasculature segmentation results?
- What are the comparative advantages of 2D versus 3D deep learning segmentation methods?
- The epidermis itself is avascular and nourished by diffusion. The superficial vascular plexus lies at the junction between the papillary and reticular dermis, roughly 0.1 to 0.4 mm beneath the surface, whereas the underlying deep vascular plexus separates the reticular dermis from the subcutaneous fat. As dermatologic OCTA typically penetrates to 1 to 1.5 mm, both plexuses are within reach of imaging. How does segmentation performance differ between superficial and deeper vasculature in the OCTA volume?
- Which vessel quantification parameters provide the most accurate assessment of segmentation quality?
- How well do the methods generalize across different dermatological conditions?

## 2 Materials and Methods

### 2.1 DERMA-OCTA Dataset

The data used in this study are described in Rotunno et al.^26^ and together with metadata are released as the open DERMA-OCTA dataset.^25^ The DERMA-OCTA collection contains 330 three-dimensional OCTA volumes acquired from 74 volunteers aged 18 to 90 years at the Medical University of Vienna. All participants provided written informed consent, and the study adhered to the Declaration of Helsinki. The study protocol and informed consent form were reviewed and approved by the Ethics Committee of the Medical University of Vienna (EK- 1246/2013). A lab-assembled research OCT platform provided high-resolution images of multiple skin sites during a single session, yielding on average four volumes per person. The OCT system was built in-house with a custom probe for patient imaging, around a commercial akinetic swept source (1310 nm center wavelength; 29 nm bandwidth). Volumes covered $1\;cm^{2}$ (512 A-lines × 512 Bscans) with 1/1.5  mm penetration in skin. The lateral and axial resolutions in air were 19.5 and 13.7  μm, respectively. A drop of distilled water was applied between the imaging window and skin for refractive-index matching. The detailed working principles of the homemade OCTA system have been described in our previous works.^7^^,^^27^^,^^28^ Of the total dataset acquired, 235 volumes come from 65 patients with CVD and span all CEAP stages C1-C6, 76 volumes originate from 13 healthy controls, and the remaining 19 volumes capture nine subjects with various malignant or benign lesions, including squamous and basal cell carcinoma, Bowen’s disease, actinic and seborrheic keratosis, and systemic sclerosis. The CVD volumes are split patient-wise into training, validation, and test sets, whereas the dermatological lesion volumes form an independent test subset.

### 2.2 OCTA Data Preprocessing

Before training the segmentation methods, a standardized preprocessing pipeline is applied to all OCTA volumes. First, each volume undergoes automatic surface detection to remove skin tilt so en-face slices align with the epidermis. Subsequently, the initial 30 Bscans that are distorted by the acquisition pattern are discarded, resulting in a 90×512×483  pixel cube. Four enhancement steps then follow in sequence:

- Bscan normalization: For each Bscan, pixel intensities are divided by that scan’s mean intensity so that the normalized image has a mean intensity of 1. Small skin motions and changes in skin–probe contact pressure and/or background cumulative noise, within an OCTA acquisition, can modulate the OCT signal of static tissue over time, leading to inter-Bscan intensity fluctuations and global brightness variations across entire OCTA Bscans. These effects shift the global brightness of entire Bscans relative to one another and manifest as vertical bright/dark stripes in enface projections and as a nonuniform background in the 3D stack. Normalizing each Bscan to unit mean suppresses global intensity shifts across the slow-scan direction, leaving relative contrasts within each Bscan unchanged. The attenuation becomes evident at the volume/enface level: the presence of vertical stripes in projections decreases, and downstream filters (e.g., 3D median) operate on a more homogeneous stack. This normalization is not a motion artifact correction algorithm per sé, but its scope is the suppression of inter-frame multiplicative bias, which can be introduced by various factors, including small movements;
- Projection artifact attenuation: a step-down exponential filter attenuates projection artifacts. This filter progressively subtracts a fraction of the overlying signal as depth increases, reducing the false appearance of deep flow while preserving real capillaries;^29^
- Filtering and contrast enhancement: a 3D median kernel together with luminance-based contrast stretching reduces noise and sharpens vessel boundaries. This would lead to a steeper grey-level slope that improves vessel/background separation;^30^
- Vesselness filtering: finally, a Frangi filter that amplifies tubular structures was implemented.^31^ A grid search was conducted over the Frangi filter parameters, varying scale ranges with different step sizes and weights α (sensitivity to plate-like structures) and β (suppression of blob-like responses). The chosen configuration with scale range [4:8], step size of 2, α=0.01, and β=1 enhanced vessels at a multiscale level, minimized noise and non-vascular responses, and suppressed sheet-like or blob-like signals.

**Fig. 1:**
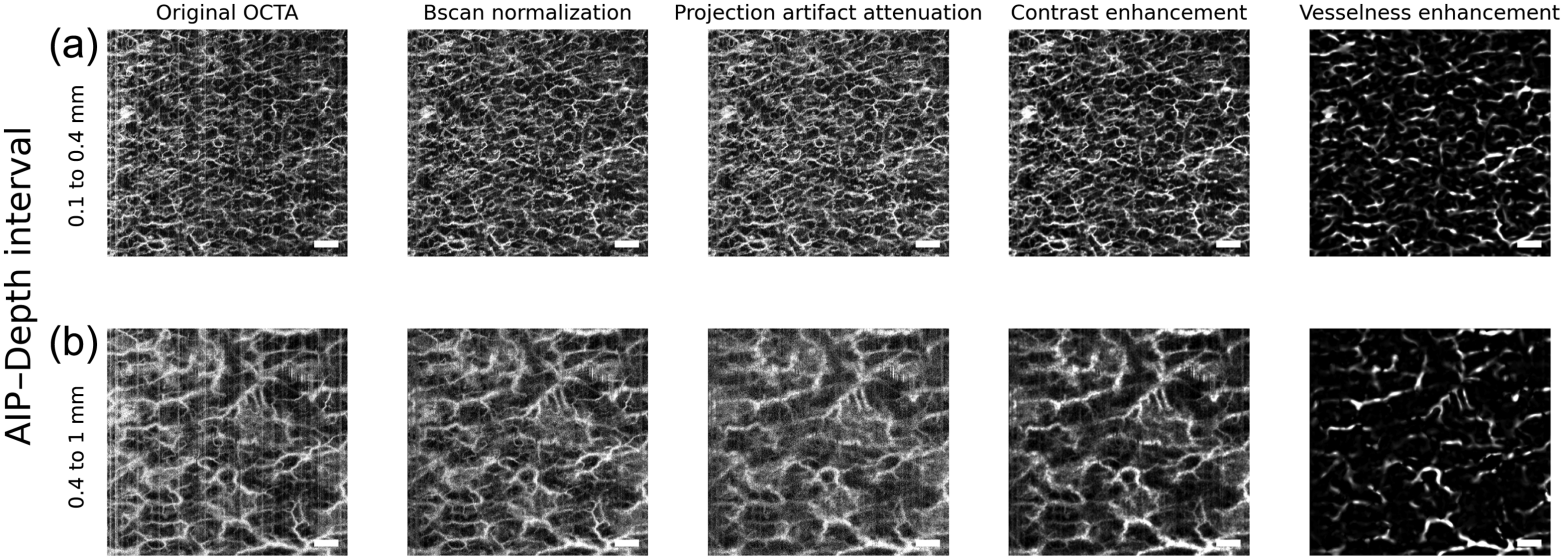
Processing steps on a representative OCTA volume. (a) Superficial AIP; (b) deep AIP. From left to right: original, normalized, projection-corrected, filtered/contrast, vessel-enhanced OCTA images. Scale bar: 1 mm.

Each preprocessing stage yields its own version of the data, and for each version, three average-intensity projections (AIP) are saved after clipping intensities at the 99th percentile and converting to 8-bit: one across the entire depth, one for superficial layers, and one for deeper layers. The AIP was utilized for en face image generation as it is less sensitive to extreme values, which can be caused by noise or artifacts when compared with the maximum intensity projection (see Fig. S1 in the Supplementary Material).^28^ Figure 1 shows an example of the effect of the described preprocessing steps.

Reference labels are produced semi-automatically in Amira (Amira 2020, Thermo Fisher Scientific Inc., Waltham, Massachusetts, United States) on the acquired volumes. Prior to labeling, volumes underwent Bscan intensity normalization and projection-artifact attenuation to enhance vessel visibility and reduce ambiguity in vessel identification. A global threshold was then applied to obtain an initial 3D mask. Using a standardized refinement protocol, annotators manually corrected the mask (removal of artifacts, interpolation of disconnected vessels), followed by 3D smoothing and removal of small, isolated components. This semi-automatic procedure provides a common starting point and consistent editing rules, thereby reducing variability between annotators. Four annotators performed the ground truth with refinements. Segmentation reliability and clinical plausibility were verified across the dataset by one technician and one clinician.

Two-dimensional ground-truth masks are then generated by projecting the superficial, deep, and full-depth 3D masks and binarizing each projection with Otsu’s method. The outcome is a set of five progressively processed 3D datasets, each paired with consistent 2D and 3D vessel annotations ready for algorithm development and benchmarking.

### 2.3 OCTA Segmentation Models

The preprocessed data were used to train, validate, and test different segmentation methods: five 2D segmentation CNN architectures using projection images and one 3D segmentation CNN architecture using the entire volumetric datasets. An additional procedure involved using the same 3D CNN architecture, followed by extrapolating 2D projections to achieve a 3D-to-2D methodology. The architectures, though based on different approaches, are well-established in the literature; this allowed for a controlled and reproducible assessment of the processing methods’ impact. It is important to note that this study’s primary objective is not to present novel segmentation methods, but rather to evaluate how various preprocessing techniques affect the performance of established CNN architectures. The processed data were split at the patient level to maintain class balance, yielding 198 acquisitions for training, 68 for validation, 45 for the standard test set, and 19 for the additional test set that contains previously unseen pathologies. Details on the training, validation, and test sets division are available in Table S1 in the Supplementary Material.

The open DERMA-OCTA dataset was integrated with the automatic segmentations obtained by each implemented method, consisting of:

- three separate 2D automatic segmentation outputs: one for the superficial layer, one for the deep layer, and one for the entire volume—per OCTA scan, processing step, and network combination.
- one 3D automatic segmentation output for each OCTA data and for each processing step,
- three separate 2D automatic segmentation outputs: one for the superficial layer, one for the deep layer, and one for the entire volume—per OCTA data extracted from the 3D automatic segmentation outputs.

#### 2.3.1 3D segmentation model

The architecture chosen to segment the 3D OCTA data was the standard 3D Unet^32^ with DenseNet169 as encoder. The network was developed on the TensorFlow platform using the Keras API. The encoder consists of four convolutional blocks with 8, 16, 32, and 64 filters, respectively, followed by a bottleneck layer containing 128 filters. The decoder mirrors the encoder with four corresponding blocks containing 128, 64, 32, and 16 filters, respectively. The final output is generated through a 3D convolutional layer with a sigmoid activation function for voxel-wise segmentation. The training employed a Dice loss and Adam optimizer with a learning rate of 0.0001 over 50 epochs. Post-processing included the removal of connected objects smaller than 100 pixels in volume.

#### 2.3.2 2D segmentation models

For a comprehensive evaluation, we implemented five different 2D segmentation models that represent the evolution of deep learning approaches in medical image segmentation over the past years. The first network tested is the Unet,^33^ a fundamental architecture that introduced skip connections between encoder and decoder paths, proving particularly effective for biomedical segmentation tasks. We then implemented DeepLabv3,^34^ which enhanced the basic segmentation approach by incorporating atrous convolutions for better multiscale processing. ConvNeXt^35^ was selected as a third model as it effectively modernizes classical CNN designs while maintaining computational efficiency. Moving towards more recent approaches, we tested K-Net,^36^ which adaptively processes features through dynamic kernel selection. The final architecture in our evaluation is the Swin Transformer,^37^ chosen for its innovative use of hierarchical transformer blocks that capture both fine details and broader contextual information.

#### 2.3.3 Evaluation metrics

The segmentation performances were evaluated on the test set by comparing automatic segmentations from different architectures against the ground truth using both standard and specialized metrics. The evaluation included the Dice coefficient, precision, recall, and balanced accuracy, which are standard pixel-level segmentation metrics that are crucial to evaluate overall segmentation performance, but they may not fully capture the complexities of vasculature network segmentation, particularly regarding network connectivity and complexity.

Three key parameters were used to extend the validation and evaluate vascular network complexity and connectivity. Let S be the predicted binary mask and $S_{gt}$ the ground-truth mask; |X| is the pixel/voxel count of set X. The parameters are then described as:

- The vascular density (VD), defined as the number of pixels/voxels belonging to the segmentation |S| divided by the total number of pixels/voxels |Ω|, providing the fraction of the field-of-view occupied by vessels:
  $$VD(S)=\frac{\left|S\right|}{\left|\Omega \right|}.$$ (1)
- The vasculature fragmentation (VF), computed as the number of connected components (#c) in the mask (8-connected in 2D, 26-connected in 3D), to give an idea of the vessel disconnections:
  VF(S)=#c(S). (2)
- The fractal dimension (FD), calculated following the box-counting method^38^ on the binary masks. Boxes of different sizes were placed in an iterative way over the 2D projection or volume. In particular, the maximum size was given as the largest power of 2 that fits in the smallest image/volume dimension; 20 scales, and 5 offsets were evaluated to select the smallest set of boxes covering all pixels/voxels > 0. The fractal dimension considers the number of boxes at different scales that contain both the vessel and the background, and it is calculated as the slope of the linear fit of log N(D) versus log D, where N(D) represents the number of boxes of side D intersecting the mask:
  $$FD=\underset{D\to 0}{\lim}(-\frac{\log\;N(D)}{\log\;D}).$$ (3)

These parameters are computed on both the automatic segmentations and the ground truth segmentations. Optimal performance is then indicated when the relative percentage difference of VD, VF, and FD between the automatic and manual segmentations approaches zero, indicating a closer alignment of the automatic results with the reference data.

Furthermore, additional metrics proposed by Giarratano et al.^39^ and Gegundez-Arias et al.^40^ were implemented to evaluate mask overlapping, fragmentation, and coincidence, which have been shown to be particularly useful for evaluating vascular network connectivity:

$$\mathrm{CALonn}(S,S_{gt})=1-\min(1,\frac{\left|\#c(S_{gt})-\#c(S)\right|}{\#S_{gt}}),$$ (4)

$$\mathrm{CALarea}(S,S_{gt})=\frac{\#((\delta_{\alpha }(S)\cap S_{gt})\cup (S\cap \delta_{\alpha }(S_{gt}))}{\#(S\cup S_{gt})},$$ (5)

$$\mathrm{CALength}(S,S_{gt})=\frac{\#((\varphi (S)\cap \delta_{\beta }S_{gt}))\cup (\delta_{\beta }(S)\cap \varphi (S_{gt}))}{\#(\varphi (S)\cup \varphi (S_{gt}))},$$ (6)

where ${\#}_{C}(S)$ and ${\#}_{C}(S_{gt})$ denote the number of connected components in the segmented image and the ground truth image, respectively. $\#S_{gt}$ represents the number of vessel pixels within the mask. The operation $\delta_{\alpha }$ refers to a morphological dilation using a disc (2D) or ball (3D) with radius α=2; φ denotes a skeletonization process (morphological thinning to 1-pixel/voxel width), and $\delta_{\beta }$ represents a morphological dilatation using a disc (2D) or ball (3D) with radius β=3. The connectivity–area–length (CAL) metric combines these three metrics through multiplication, with 1 indicating ideal prediction and 0 indicating complete failure.

All metrics were employed for both 2D and 3D outputs.

### 2.4 Statistical Analysis

The statistical evaluation proceeded in two steps. First, a Kolmogorov–Smirnov test was conducted to assess the normality of the metric distributions. Subsequently, a one-way ANOVA analysis was performed to identify which metrics most effectively differentiated between the 2D and 3D segmentation models and various preprocessing steps. Statistical testing was conducted in two directions: across the five preprocessing steps for each architecture and across the six tested architectures for each preprocessing step. This bi-directional analysis enabled a comprehensive evaluation of both preprocessing impact and architectural performance differences.

## 3 Results

### 3.1 Performance Between Models and Processing Methods

The performance of the architectures in 2D and 3D across the five preprocessed datasets was evaluated both qualitatively and quantitatively. An accurate segmentation is expected to bypass OCTA artifacts while preserving vessel morphology and continuity at different scales. This translates into higher values for the standard segmentation metrics and the CAL metrics, alongside values close to zero for the remaining specific metrics calculated (i.e., relative percentage difference of VD, VF, and FD). For simplicity, in the figures and table below, only the Dice and CAL metrics are presented (ANOVA p-values<0.05). In this manner, both a standard metric for generic segmentation tasks and a specific metric for vascular segmentation are highlighted. The results over all the metrics are provided in Sec. 3 and Table S2 in the Supplementary Material.

**Fig. 2:**
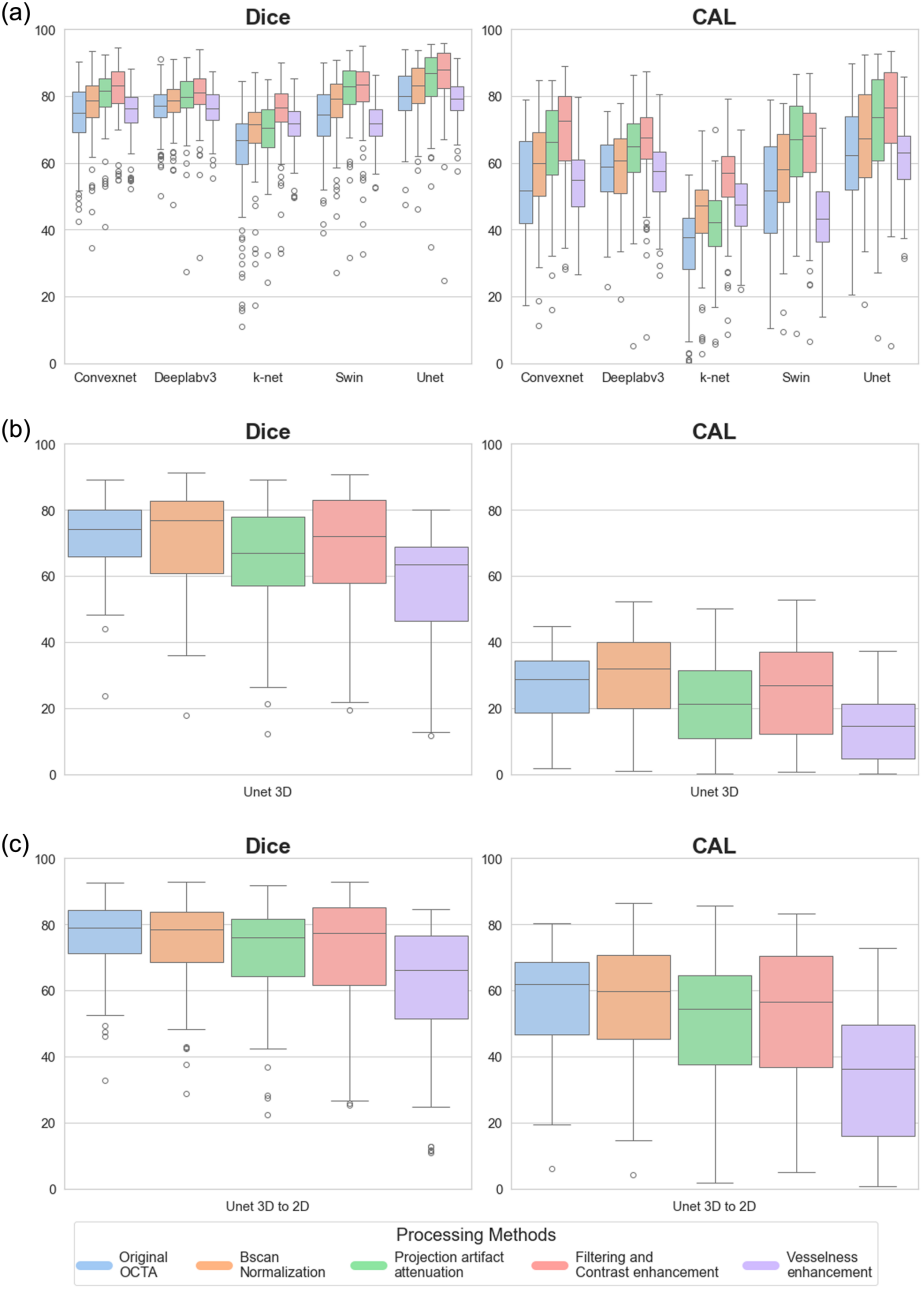
Quantitative comparison of segmentation performance across different approaches and preprocessing methods. Box plots showing the distribution of Dice coefficient (left) and CAL metric (right) for: (a) 2D models, (b) 3D models, and (c) 3D-to-2D approach (metrics calculated on AIP extracted from 3D predictions). Different colors indicate different preprocessing methods, as shown in the legend.

To numerically appreciate the performance trends in the datasets and across the models, a combined graph is reported. Figure 2 shows the performance distribution across the preprocessing steps for 2D models [Fig. 2(a)], the 3D model [Fig. 2(b)], and the intermediate approach from 3D-to-2D [Fig. 2(c)]. In the 2D setting, both Dice and CAL metrics showed progressive improvement through the preprocessing pipeline until the filtering and contrast enhancement stage. However, a notable performance decline was observed after implementing vesselness filtering. While similar trends were observed in the 3D approach, the effects were less pronounced. The 3D-to-2D approach showed significant advantages over direct 3D segmentation, achieving a CAL mean value of 57.64%±15.02 for projections extracted from 3D predictions compared to 26.77%±10.55 for raw 3D data segmentation.

Regarding computational requirements, the 2D Unet processes a single volume in ∼90  s on a GPU (NVIDIA RTX 3090) and 15 min on a CPU. The 3D Unet requires ∼1  min per volume on GPU and becomes impractical on CPU, with processing times exceeding 45 min. The more complex architectures (ConvNeXt, K-Net, Swin Transformer) introduce additional overhead ranging from 30% to 100% in inference time compared to Unet, particularly in CPU environments, without providing substantial performance benefits. This computational burden makes them less suitable for clinical deployment, especially in settings without dedicated GPU hardware.

**Fig. 3:**
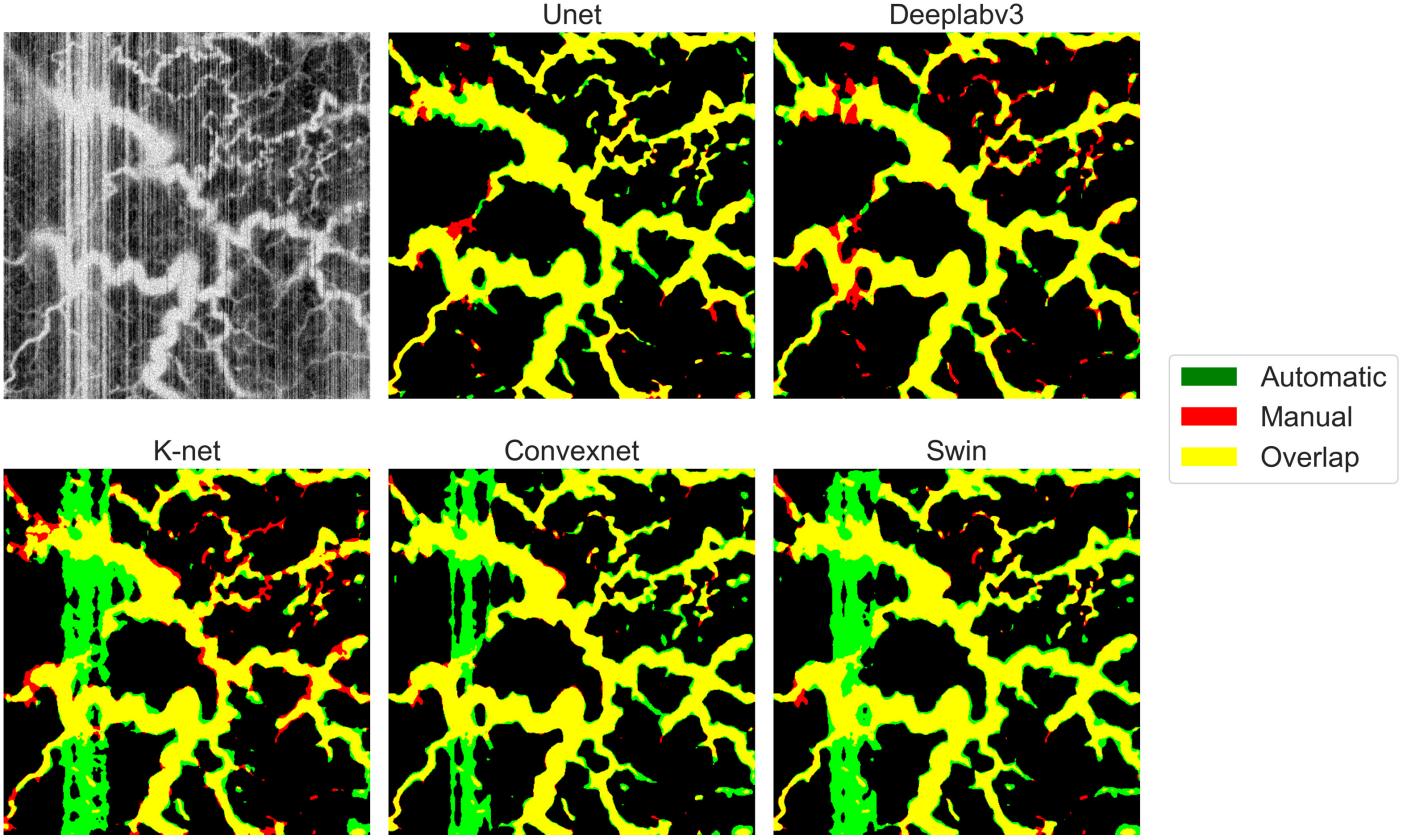
Qualitative comparison of 2D segmentation architectures on an example OCTA AIP image. The figure shows the original OCTA AIP with a notable vertical artifact in the left portion and the results provided by the segmentation networks. Only automatic predictions are shown in green, only ground truth segmentations is shown in red. Perfect overlap appears in yellow. Ground truth segmentation video: (Video 1, MPG, 44.8 MB [URL: https://doi.org/10.1117/1.JBO.30.11.116005.s1]).

To evaluate the performance of different 2D architectures, a qualitative comparison was also conducted. Figure 3 presents OCTA AIP examples with their corresponding automatic segmentations, where overlapping manual and automatic predictions are shown in yellow, along with only automatic predictions (green) and only manual ground truth masks (red). The full ground-truth creation process is illustrated in Video 1. Among the tested architectures, Unet and Deeplabv3 demonstrated superior capabilities in artifact recognition and avoidance. Particularly noteworthy was their ability to correctly handle the intense white vertical artifact lines present in the left portion of the OCTA image. However, Unet showed better performance in maintaining segmentation continuity, successfully identifying vascular structures obscured by artifacts, while Deeplabv3 often missed smaller vessel segments.

**Fig. 4:**
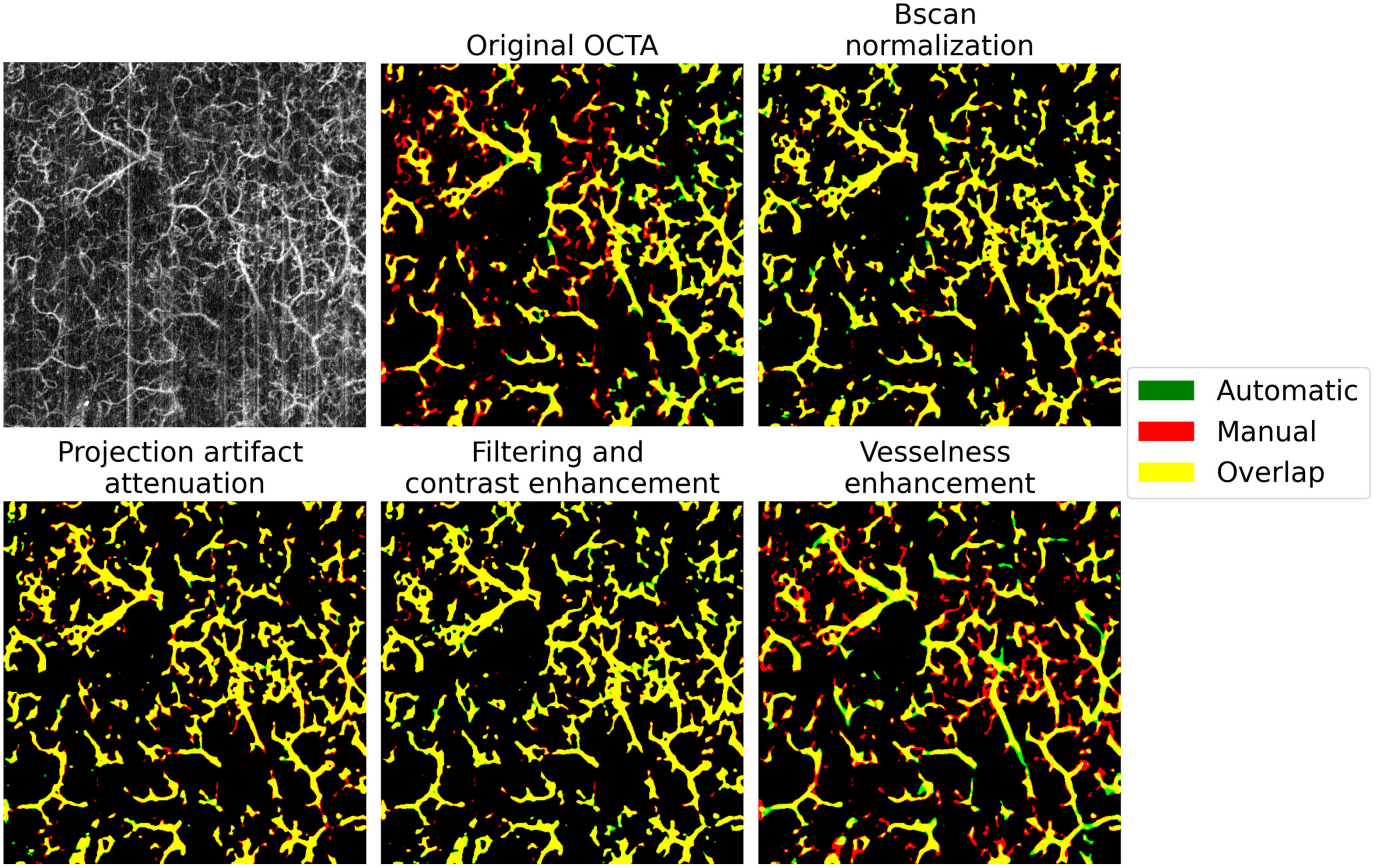
Impact of different preprocessing methods on Unet segmentation performance. Automatic predictions (green) are overlaid with ground truth masks (red). The overlap between the two segmentations is shown in yellow.

Given the Unet’s superior overall performance, a detailed analysis of the preprocessing impact on this architecture was conducted. Figure 4 illustrates OCTA AIP examples processed through different preprocessing stages and segmented using the Unet network. The baseline Unet, trained on original OCTA data, showed limitations in detecting fine vessel details due to poor contrast. The Bscan normalization and projection artifact attenuation preprocessing steps showed intermediate performance - while not achieving optimal contrast, they effectively highlighted relevant vascular information and successfully suppressed artifacts. Significant improvements were observed with the filtering and contrast enhancement dataset, enabling successful segmentation of even smaller vessels. However, the vesselness enhancement preprocessing led to mixed results, introducing false vessel connections and showing inconsistencies in handling vessels of different radii.

### 3.2 Performance between Superficial and Deep Layers

**Fig. 5:**
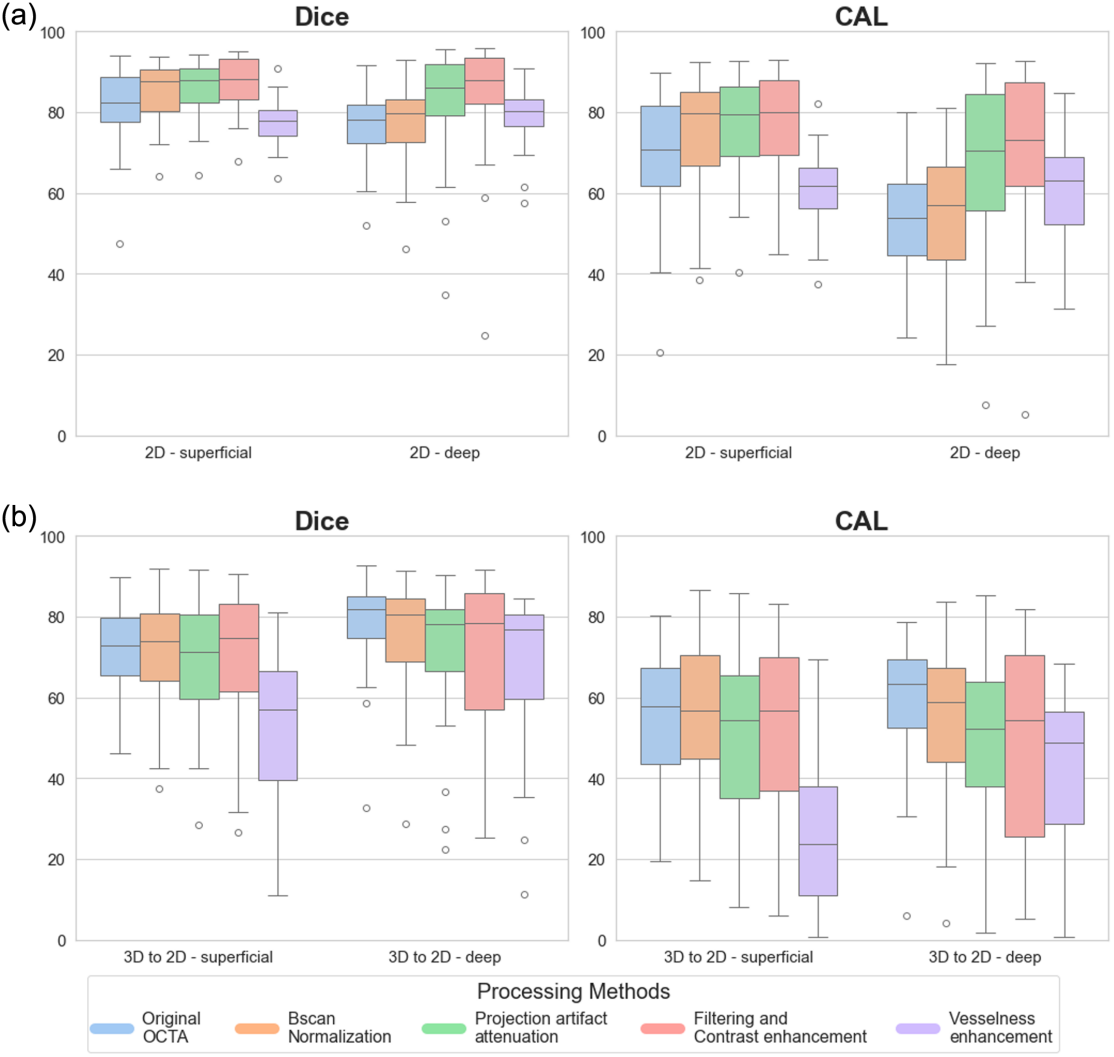
Quantitative comparison of segmentation metrics (Dice coefficient and CAL) between (a) 2D Unet model and (b) 3D-to-2D approach analyzed across different skin depths. Results are separated into superficial and deep layers and further grouped by preprocessing methods.

To evaluate the effectiveness of 2D versus 3D approaches in segmenting different skin depths, a comparative analysis was performed, separating superficial and deep layers. Figure 5 depicts the quantitative metrics distribution for both 2D and 3D Unet models across different preprocessing methods. For a fair comparison, the 3D results are reported using the 3D-to-2D approach. The 2D approach demonstrated superior performance in superficial layers, where vessels typically show better contrast and definition. Conversely, the 3D approach achieved more consistent results in both the superficial and deeper layers, with a slight performance increase at the deeper layer, likely due to its ability to leverage volumetric context for identifying larger, less distinct vessels. Both approaches showed decreased performance with vesselness enhancement preprocessing, suggesting this step might not be optimal for layer-specific segmentation.

**Fig. 6:**
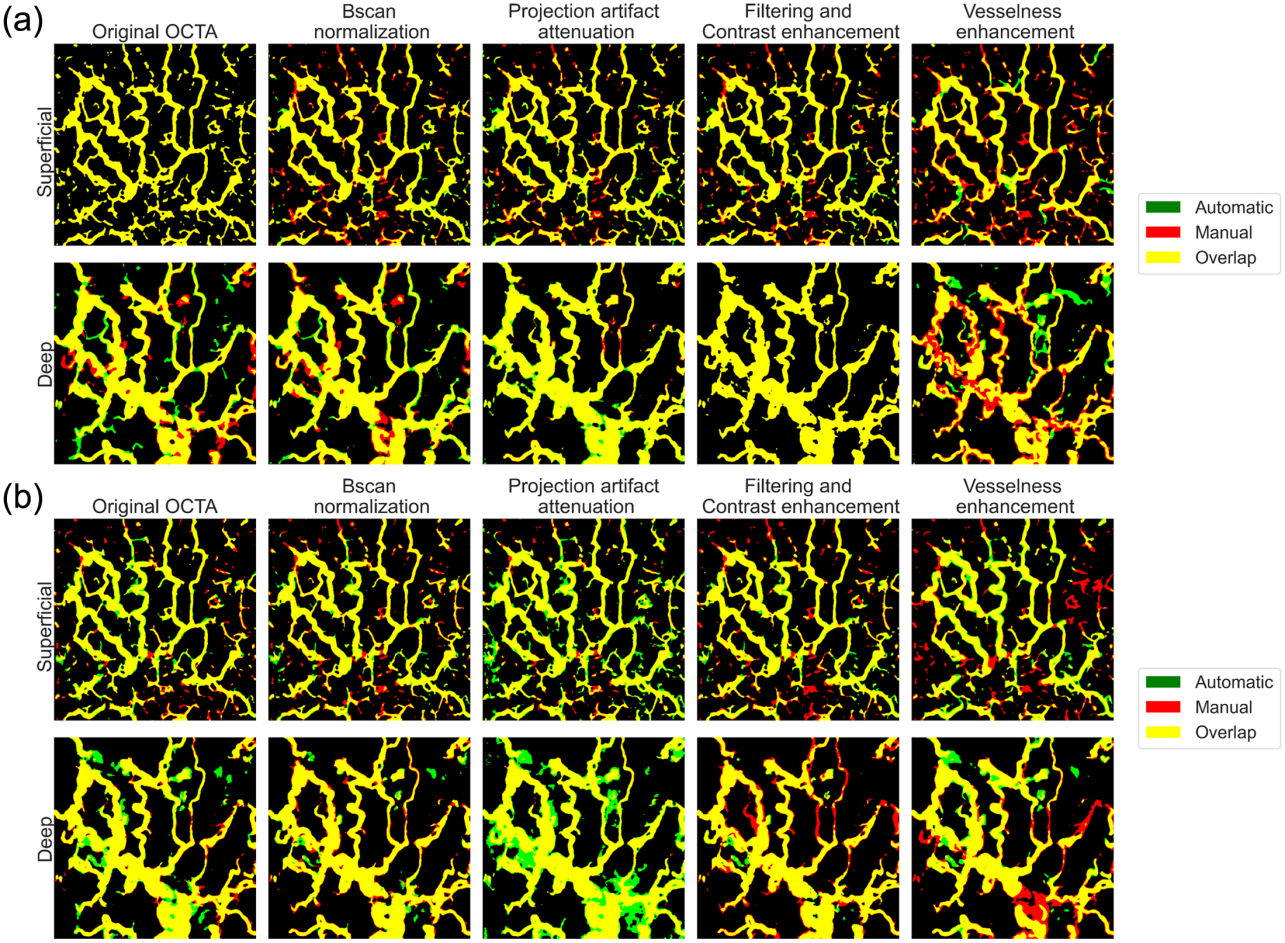
Output from different processing steps on the 2D (a) and 3D (b) Unet model and grouped by the superficial and deep layer segmentation outputs. The ground truth is colored in red, the network prediction is colored in green, and the overlapping area is colored in yellow.

The qualitative analysis in Fig. 6 provides visual confirmation of these quantitative findings. In superficial layers, where fine and complex vessel patterns dominate, the 2D Unet [Fig. 6(a)] showed better capability in preserving detailed structures compared to its 3D counterpart. The overlap between the automatic and manual predictions (shown in yellow) demonstrates higher accuracy in vessel detection and better preservation of small vessel continuity in the 2D approach for superficial regions.

Conversely, in deeper layers characterized by larger vessel structures, the 3D Unet [Fig. 6(b)] showed improved performance in correctly identifying vessel patterns. This advantage was particularly evident in areas affected by projection artifacts, where the 3D approach’s ability to utilize volumetric information helped in distinguishing genuine deep vessels from projection artifacts.

### 3.3 Performance on the Test Set from Different Pathologies

To evaluate the versatility and generalizability of the proposed segmentation approaches, we tested our models on OCTA volumes acquired from patients with different pathologies. Although our training set included data from patients with CVD, the test set from different pathologies comprised OCTA scans from patients with malignant and benign skin lesions, which typically present distinct vascular patterns.

Table 1 compares the performance of both 2D and 3D Unet implementations across the original test set (CVD patients) and the test set from different pathologies (skin lesions). The complete metrics for all tested architectures on the different pathologies test set are provided in Sec. 3, Tables S3–S5, and Figs. S2–S4 in the Supplementary Material. The results show that although the relative performance trends across preprocessing methods remain consistent between the two test sets, there is an expected decrease in absolute performance metrics when testing on different pathologies. The 2D Unet maintained better performance compared with its 3D counterpart across both test sets, with contrast enhancement preprocessing yielding the best results (Dice: 86.34±8.83 versus 70.05±15.30 for original versus different pathologies). Notably, vesselness enhancement showed particularly poor performance on the different pathologies test set, suggesting this preprocessing method might be less robust to variations in vascular patterns.

**Table 1:** Performance of the 2D and 3D Unet model tested on the test set from different pathologies.

			Original OCTA	Bscan normalization	Projection artifact attenuation	Contrast enhancement	Vesselness enhancement
Unet 2D	**Test set**	**Dice**	79.62 ± 8.45	81.94 ± 8.40	84.56 ± 9.07	**86.34 ± 8.83**	79.05 ± 5.68
		**CAL**	62.19 ± 15.13	66.39 ± 15.98	71.41 ± 16.00	**74.59 ± 14.50**	61.63 ± 10.45
	**Test set from different pathologies**	**Dice**	56.70 ± 17.04	59.34 ± 16.55	66.23 ± 16.25	**70.05 ± 15.30**	22.18 ± 10.53
		**CAL**	35.68 ± 18.40	39.31 ± 18.30	48.18 ± 19.89	**54.41 ± 19.48**	6.15 ± 6.10
Unet 3D	**Test set**	**Dice**	71.32 ± 12.69	**71.14 ± 16.46**	64.20 ± 18.08	66.70 ± 18.64	57.23 ± 16.36
		**CAL**	26.77 ± 10.55	**28.68 ± 14.31**	21.88 ± 13.68	25.06 ± 15.45	14.09 ± 9.68
	**Test set from different pathologies**	**Dice**	56.42 ± 15.08	**55.79 ± 15.37**	48.99 ± 15.32	53.59 ± 16.99	38.05 ± 17.61
		**CAL**	17.83 ± 9.82	16.96 ± 10.37	12.96 ± 10.51	**18.00 ± 12.76**	8.39 ± 8.44

Note: bold values indicate the best performance for each row.

## 4 Discussion

In this study, we explored the challenges and advancements in OCTA-based vessel segmentation for dermatological applications using the published DERMA-OCTA dataset. Our results highlight the complexity of accurately segmenting vasculature in skin tissue, particularly due to the heterogeneous nature of the skin, varying vessel architectures, and the presence of artifacts.

How do different preprocessing methods impact deep learning vasculature segmentation results? The employed 2D and 3D deep learning models demonstrated promising performance, and our findings underline the fact that preprocessing techniques do indeed have an impact on achieving accurate vessel delineation for dermatological applications. Although the Bscan normalization, projection artifact attenuation, and filtering and contrast enhancement steps showed similar outcomes, each successive step provided a slight gradual improvement in performance. Notably, vesselness enhancement resulted in a loss of vascular network morphology and a significant decline in all metrics. The poor performance of the vesselness enhancement can be attributed to the Frangi vesselness filter’s limitations. Indeed, although effective for enhancing blood vessels, the filter’s performance is highly dependent on the choice of scales, which may not always align with the varying vessel sizes in an image, leading to missed detections. The filter also typically assumes vessels are tubular and elongated, which makes it less effective at handling bifurcations, junctions, or irregularly shaped vessels, which can be present in the vasculature of different skin lesions. It can also struggle with noise and low vessel-to-background contrast, sometimes enhancing nonvascular features or producing false positives. The method requires careful tuning of parameters, which can be challenging to generalize across different lesions, which present varying vasculature complexities.^41^ It should be noted that the manual segmentations were created on the projection artifact attenuated datasets rather than the vesselness-enhanced datasets, potentially influencing the results. Although recent studies^42^ have investigated new techniques to improve vesselness structures, these were primarily focused on other imaging modalities, leaving the dermatological application of OCTA relatively unexplored.

What are the comparative advantages of 2D versus 3D deep learning segmentation methods? Among the tested 2D deep learning architectures, Unet demonstrated superior performance, achieving the highest metrics and lowest standard deviations (Fig. 2). Its encoder–decoder structure with skip connections effectively preserves fine details while capturing contextual information, making it ideal for vessel segmentation. By contrast, K-Net underperformed likely due to its focus on instance-level discrimination rather than pixel-wise classification. Deeplabv3 performed well but struggled with fine vessel reconstruction mainly due to its single-stage upsampling. ConvNeXt and the Swin Transformer showed competitive results but faced challenges with small, thin vessels. Overall, the Unet’s ability to balance feature extraction and spatial precision proved most effective for this task. The 3D Unet model, while computationally expensive, showed similar trends to 2D results but with lower baseline performance. This performance gap might be attributed to the larger training dataset available for 2D models compared with 3D volumes. Future improvements in 3D segmentation architectures and computational resources could better leverage spatial continuity within the 3D data. Moreover, it is crucial to underline how obtaining accurate 3D manual segmentations of vasculature is a challenging and time-consuming task due to the complex and intricate nature of vascular structures. Blood vessels often exhibit varying diameters, tortuous paths, and branching patterns, making it difficult to delineate them consistently across all dimensions. Low contrast between vessels and surrounding tissues, coupled with imaging artifacts and noise, further complicates the process. In addition, small or thin vessels may be poorly resolved, leading to incomplete or inaccurate annotations. To mitigate some of these challenges, projecting 3D data into 2D representations can simplify the segmentation process by reducing the complexity of the structure and improving visualization. However, this comes at the cost of losing depth information, which is crucial for fully understanding the vascular network in 3D.

How does segmentation performance differ between superficial and deep vasculature? Indeed, the analysis of depth-dependent performance revealed that 2D models struggled more with accurately segmenting vessels in deeper structures, whereas the 3D Unet, despite producing slightly worse overall results, maintained more consistent performance across both superficial and deeper layers. Indeed, the 3D architecture’s ability to process volumetric data allowed it to maintain a more robust segmentation capability across varying depths. The 3D Unet leverages the spatial relationships between adjacent slices, allowing it to capture the full context of vascular structures and mitigate the challenges of depth-related ambiguities that 2D models face. Performance differences between preprocessing steps are much more subtle with the 3D Unet, and interestingly, a slight increase can be observed when less preprocessing is applied.

Which vessel quantification parameters provide the most accurate assessment of segmentation quality? To measure the quality of the segmentation, standard metrics and specific vessel quantification parameters were calculated, with a total of 11 metrics extracted from each dataset. The obtained results suggest that standard metrics (i.e., Dice, precision, recall, balanced accuracy) are not always accurate for assessing the segmentation of vascular structures. In particular, the CAL metric, which combines information on the degree of overlap, fragmentation, and coincidence between the automatic and the predicted mask and has been proposed and evaluated mainly for ophthalmological applications, proved to be particularly valuable also in the dermatology application setting.

How well do the methods generalize across different dermatological conditions? The results reported in this study highlight the challenges of generalizing deep-learning-based vessel segmentation models across different dermatological conditions that present very different vascular patterns. Although both 2D and 3D Unet architectures demonstrated consistent performance trends across preprocessing steps, testing on OCTA scans from patients with different pathologies, including malignant and benign skin lesions, led to a decrease in absolute performance metrics. These findings suggest that to develop a generalizable vessel segmentation method for dermatological OCTA, it is crucial to incorporate a broad range of pathologies during training. By exposing models to the variability in vascular patterns across different skin conditions, model architectures can be better optimized for diverse clinical applications.

**Fig. 7:**
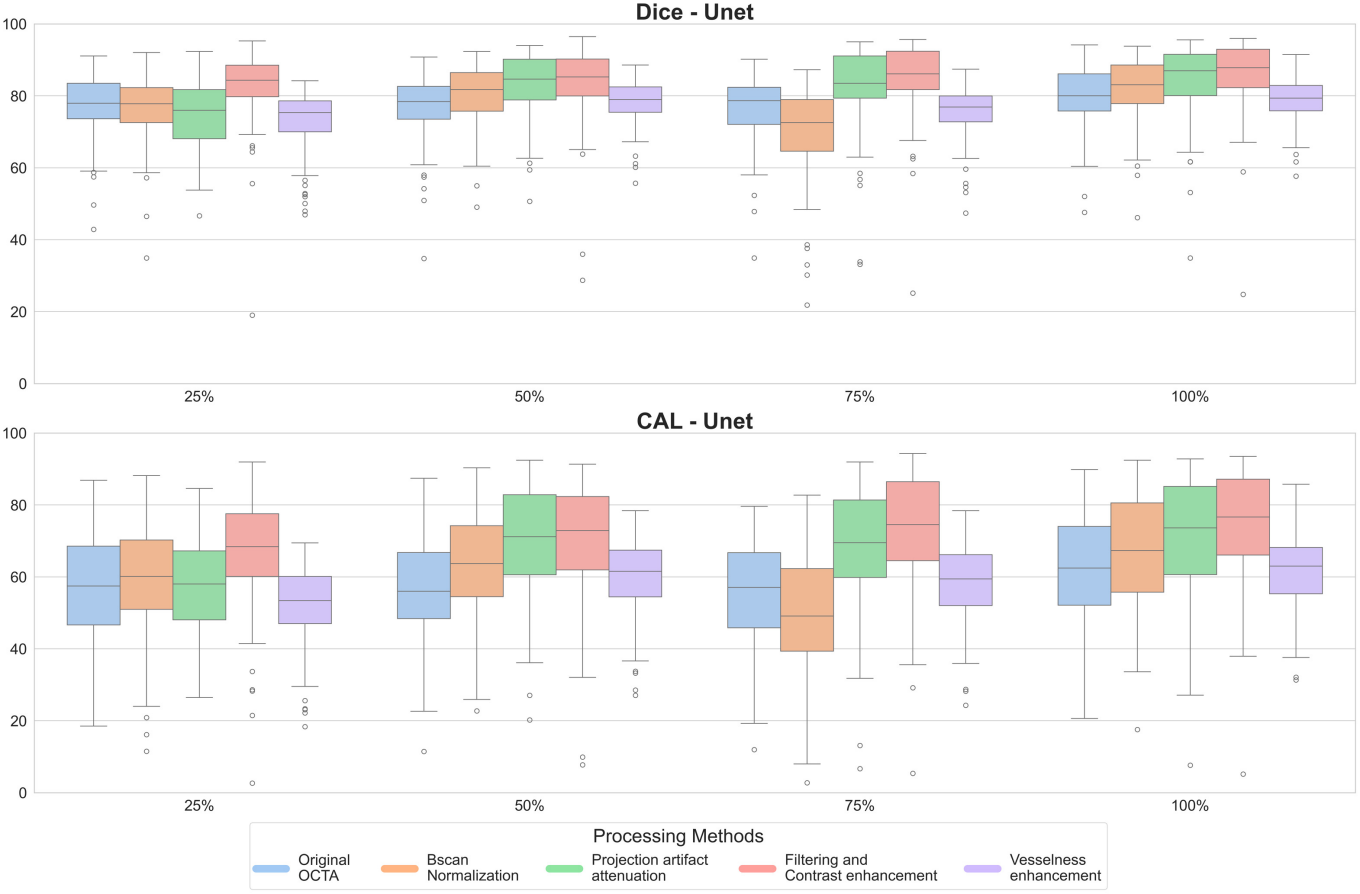
Dice score and CAL metric comparison across preprocessing methods and training set sizes (25%, 50%, 75%, 100% of the original training set). Different colors represent the preprocessing methods, as indicated in the legend.

Although our study provides valuable insights into vessel segmentation for dermatological OCTA, there are several limitations to consider. First, the deep learning model architectures we tested, including the 2D architectures and 3D Unet, do not represent the optimal solution and the most recent model architecture. In addition, the DERMA-OCTA dataset, the first open-access dermatological OCTA collection, is limited in size and diversity, with a relatively small cohort of patients and a focus on specific pathologies. This limitation may affect the generalizability of our findings across a broader range of dermatological conditions. It is important to note, however, that the primary goal of our study was not to present a novel segmentation method but to highlight key considerations for dermatological OCTA, with a focus on the impact of preprocessing techniques. Indeed, our findings show how a preprocessing pipeline that includes Bscan normalization, projection artifact attenuation, and contrast enhancement, paired with a simple 2D Unet segmentation method, provides promising results. To further assess the influence of dataset size on segmentation performance, the training set was progressively reduced to 25%, 50%, and 75% of its original size, while the validation and test sets were kept unchanged (see Table S6 in the Supplementary Material). Figure 7 shows the results obtained for the Dice coefficient and the CAL measure. Interestingly, even with a greatly reduced dataset used for training, the trend of the preprocessing methods that show the best performance with contrast enhancement and a drop when including the vesselness filtering is still clearly observable. The trend is repeatable across all dataset sizes, and it can be appreciated how the basic Unet results show baseline performance results, which are consistently higher than the other considered methods (see Fig. S5 in the Supplementary Material). These additional results further support the robustness of our findings and the proposed preprocessing pipeline. Indeed, we aim to provide a foundation for future work that can further refine segmentation models and preprocessing strategies to better address the complexities of dermatological OCTA. In the future, we aim to further enhance the open dataset and explore other methods using multiple OCT devices to create a new multidevice pipeline offering new opportunities for model development, benchmarking, and the advancement of OCTA-based dermatological diagnostics.

## 5 Conclusion

This study provides a comprehensive evaluation of OCTA vessel segmentation approaches for dermatological applications. Our analysis demonstrates that careful preprocessing of OCTA data significantly impacts segmentation accuracy, with contrast enhancement proving most beneficial while vesselness filtering potentially degrading performance. Among the tested architectures, the 2D Unet emerged as the most effective solution, particularly for superficial vessel segmentation, whereas 3D approaches showed advantages in maintaining performance results across different depths.

Specifically, we recommend:

• a preprocessing pipeline that includes Bscan normalization, projection artifact attenuation, and contrast enhancement, but avoids vesselness filtering;
• the use of 2D Unet for general vessel segmentation tasks, with consideration of 3D approaches when deep tissue analysis is crucial;
• the adoption of specialized vessel metrics, particularly the CAL index, alongside traditional segmentation metrics for comprehensive evaluation.

These findings establish a practical foundation for implementing OCTA vessel segmentation in dermatological applications while also highlighting areas requiring further research, particularly in improving generalization across different pathologies and optimizing 3D segmentation approaches.

## Supplementary Material

10.1117/1.JBO.30.11.116005.s01

10.1117/1.JBO.30.11.116005.s1

## Data Availability

The data and the codes presented in this article are publicly available in Zenodo.^25^
