# Supplementary material for: Impact of image preprocessing on dermatological OCTA vessel segmentation: a DERMA-OCTA study
Source: J Biomed Opt. 2025 Nov 24;30(11):116005. doi: 10.1117/1.JBO.30.11.116005 (PMC12643383; doi:10.1117/1.JBO.30.11.116005)
Supplement: Supplementary file 1 [file JBO_030_116005_SD001.pdf]

SUPPLEMENTARY MATERIAL

Impact of Image Preprocessing Effects on Dermatological OCTA Vessel Segmentation: a DERMA-OCTA Study

Giulia Rotunno,[a,*] Massimo Salvi,[a,*] Julia Deinsberger,[b] Lisa Krainz,[c] Lukasz Bugyi,[c] Benedikt Weber,[b] Christoph Sinz,[d] Harald Kittler,[b] Leopold Schmetterer,[c,e,f,g,h,i,j,k] Wolfgang Drexler,[c] Mengyang Liu[c,e] and Kristen M. Meiburger,[a,]**

[a]PolitoBIOMed Lab, Department of Electronics and Telecommunications, Politecnico di Torino, Torino, Italy
[b]Department of Dermatology, Medical University of Vienna, Vienna, Austria
[c]Center for Medical Physics and Biomedical Engineering, Medical University of Vienna, Vienna, Austria
[d]Melanoma Institute Australia, The University of Sydney, Sydney, New South Wales, Australia
[e]SERI-NTU Advanced Ocular Engineering (STANCE) Program, Singapore, Singapore
[f]Singapore Eye Research Institute, Singapore, Singapore
[g]Academic Clinical Program, Duke-NUS Medical School, Singapore, Singapore
[h]School of Chemistry, Chemical Engineering and Biotechnology, Nanyang Technological University, Singapore, Singapore
[i]Department of Clinical Pharmacology, Medical University of Vienna, Vienna, Austria
[j]Fondation Ophtalmologique Adolphe De Rothschild, Paris, France
[k]Aier Hospital Group, Changsha, China

Abstract

Significance: Optical coherence tomography angiography (OCTA) offers dye-free, three-dimensional views of skin microvasculature, yet progress in developing reliable and quantitative solutions for vessel architecture analysis is slowed by heterogeneous preprocessing practices, scarce annotated data and limited evaluation metrics.

Aim: The work assesses how typical OCTA preprocessing steps influence the accuracy of deep-learning vessel segmentation and identifies network designs and metrics best suited to OCTA dermatological data.

Approach: Experiments use the open DERMA-OCTA dataset containing 330 volumes from different skin conditions; each volume is additionally provided in five progressively pre-processed versions: original, Bscan normalization, projection artifact attenuation, contrast enhancement and vesselness filtering. Segmentation is performed with representative 2D and 3D deep learning approaches. Besides standard segmentation metrics, evaluation includes the Connectivity-Area-Length (CAL) index, which proved particularly effective for assessing dermatological vessel segmentation.

Results: The analysis shows that Bscan normalization, projection artifact attenuation, and contrast enhancement incrementally improve segmentation accuracy, while vesselness enhancement can impair segmentation performance. Among the tested architectures, 2D models achieved the highest segmentation performance, although 3D approaches proved more effective for deeper tissue layers. Testing across different pathologies revealed challenges in model generalization to varied vascular patterns.

Conclusions: Combining 2D and 3D models and using topology-aware indices provides a full, clinically relevant evaluation of algorithm performance.

Keywords: Skin imaging; OCTA; Segmentation; Deep Learning; Vessel analysis.

* These authors contributed equally.
** Kristen M. Meiburger**,** E-mail: kristen.meiburger@polito.it

1. Comparison between maximum intensity projection (MIP) and average intensity projection (AIP) images

For each preprocessing stage, three average-intensity projections (AIP) are saved after clipping intensities at the 99th percentile and converting to 8-bits: one across the entire depth, one for superficial layers, and one for deeper layers. The AIP was utilized for en face image generation, as it is less sensitive to extreme values which can be caused by noise or artifacts when compared with the maximum intensity projection, which is shown here in Figure S1.

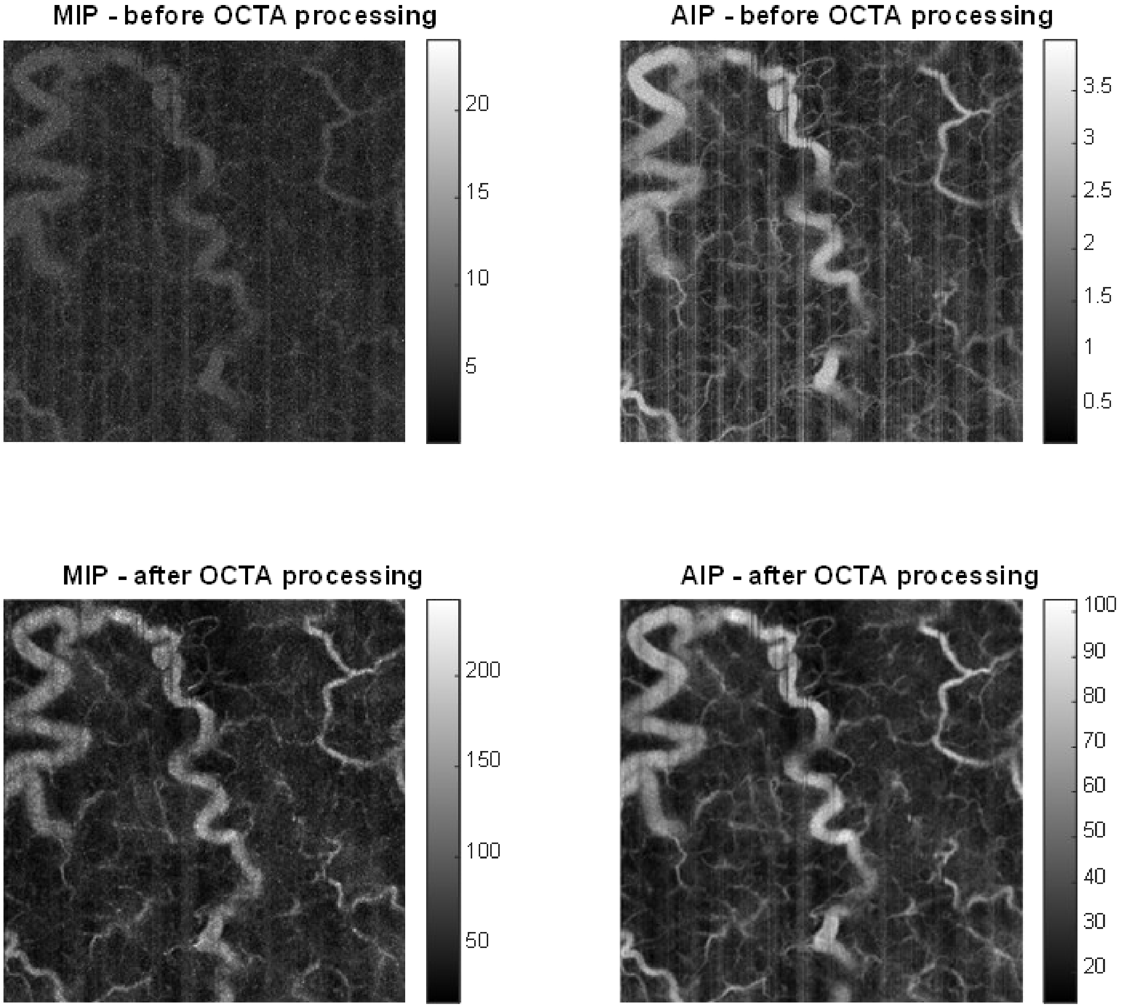

Fig. S1 Quantitative comparison between maximum intensity (MIP) and average intensity (AIP) projections for a representative OCTA volume. Columns: MIP (left), AIP (right). Rows: before processing (top), after processing (bottom).

2. Dataset division into training, validation, and test sets

Table S1 reports the complete dataset division between the various sets (training, validation, test) and the various pathologies/healthy subjects.

Table S1 Acquired data volumes divided into train, validation, test set and test set from different pathologies.

# OCTA	Healthy	CVD C1	CVD C2	CVD C3	CVD C4a	CVD C4b	CVD C4c	CVD C5	CVD C6	Tot
Training set	53	7	34	37	26	5	14	2	20	**198**
Validation set	12	4	9	13	7	4	10	2	7	**68**
Test set	11	1	3	6	7	5	5	3	4	**45**

# OCTA	SCC	Seborrheic keratosis	Morbus Bowen	Actinic keratosis	BCC	Systemic sclerosis	Tot
Test set from different pathologies	3	1	4	4	4	3	**19**

3. Complete performance evaluation metrics

A one-way ANOVA analysis was performed to identify which metrics most effectively differentiated between the 2D and 3D segmentation models and various preprocessing steps. Statistical testing was conducted in two directions: across the five preprocessing steps for each architecture, and across the six tested architectures for each preprocessing step. This bi-directional analysis enabled comprehensive evaluation of both preprocessing impact and architectural performance differences. Table S2 reports the results over all of the metrics.
Further evaluation metrics were implemented. In particular, the performance results of the 2D models and the 3D Unet model (evaluated on the test set from different pathologies) in terms of Dice, precision, recall and balanced accuracy are reported in Table S3. Table S4 reports the results in terms of Vascular density (VD), vasculature fragmentation (VF) and fractal dimension (FD). Table S5 reports the results in terms of the CAL metrics.
The results are also reported as boxplots in Figures S2 (Dice, precision, recall and balanced accuracy), S3 (VD, VF, and FD), and S4 (CAL).

Table S2 ANOVA results comparing the models and the processing steps tested.

	Unet	*Deeplabv3*	*K-net*	*Convexnet*	*Swin*	*Original OCTA*	*Bscan norm.*	*Projection artifact attenuation*	*Filtering & Contrast enhancement*	*Vesselness enhancement*	*3D Unet*
**Dice**	**5.78e-39**	**2.51e-25**	**3.07e-42**	**5.88e-21**	**1.18e-28**	**1.01e-16**	**3.8e-13**	**2.77e-14**	**3.09e-14**	**8.33e-10**	**0.000355**
**Precision**	**8.73e-38**	**2.27e-15**	**6.2e-09**	**6.13e-10**	**2.23e-19**	**1.37e-10**	**0.000622**	**0.000865**	**4.53e-10**	**1.61e-12**	**4.45e-06**
**Recall**	**1.05e-16**	**2.98e-22**	**7.57e-73**	**3.04e-21**	**9.25e-35**	**6.4e-12**	**6.26e-19**	**9.32e-24**	**4.73e-19**	**0.0144**	**9.06e-05**
**Bal. acc.**	**7.13e-42**	**1.57e-33**	**1.08e-73**	**5.67e-29**	**9.44e-36**	**2.1e-20**	**7.72e-25**	**1.88e-29**	**1.58e-22**	**0.000619**	**0.00014**
**VD**	**1.12e-07**	**0.00092**	**5.02e-09**	**0.0124**	**4.94e-17**	**0.000496**	0.0676	**0.0035**	**5.43e-08**	**2.39e-07**	**0.00233**
**VF**	**1.84e-05**	**3.17e-14**	**8.48e-11**	**7.78e-07**	**1.6e-05**	0.625	**0.003**	**3.92e-05**	**6.85e-05**	**0.00242**	**0.46**
**FD**	**2.38e-09**	**0.00026**	**2.66e-19**	**0.0157**	**3.5e-21**	**6.62e-05**	**0.017**	**0.000208**	**2.63e-08**	**1.29e-05**	**0.00623**
**CAL area**	**9.33e-38**	**1.83e-20**	**2.66e-42**	**3.38e-15**	**3.52e-31**	**5.54e-16**	**1e-11**	**1.13e-12**	**4.14e-13**	**4.42e-11**	**1.5e-05**
**CAL conn**	**0.0097**	**0.000266**	0.179	**0.00484**	**0.0409**	0.0718	**0.00724**	**0.00755**	0.0746	0.931	**0.0175**
**CAL length**	**1.42e-56**	**4.51e-36**	**1.31e-75**	**5.66e-31**	**3.61e-48**	**3.99e-28**	**1.78e-25**	**2.28e-26**	**5.44e-25**	**9.96e-18**	**2.21e-06**
**CAL**	**1.21e-49**	**1.55e-32**	**3.94e-65**	**1.75e-28**	**2.9e-46**	**1.35e-25**	**1.19e-22**	**4.46e-24**	**4.65e-24**	**1.29e-18**	**1.27e-06**

Table S3 Performance of the 2D and 3D Unet model tested on the test set from different pathologies. Dice, precision, recall and balanced accuracy are reported.

**Net**	**Dataset**	**Dice**		**Precision**		**Recall**		**Bal. Acc.**	
		**mean**	**std**	**mean**	**std**	**mean**	**std**	**mean**	**std**
**Convexnet**	**Original OCTA**	63,41	15,06	54,02	18,73	84,05	14,76	83,05	8,20
	**Norm**	65,76	15,03	58,76	19,62	81,25	15,78	83,57	8,53
	**Proj art**	66,57	14,72	59,59	19,15	81,29	15,50	83,90	8,49
	**Contrast**	68,10	14,85	60,80	18,18	82,26	16,19	84,67	8,83

	**Vesselness**	41,40	14,25	27,19	11,61	**98,80**	**4,60**	63,15	6,39
**Deeplabv3**	**Original OCTA**	55,92	15,60	42,06	15,77	91,38	11,72	78,95	7,46
	**Norm**	59,95	14,67	47,92	16,15	86,47	12,80	81,11	7,70
	**Proj art**	63,75	13,64	60,48	18,37	72,18	15,28	80,36	8,42
	**Contrast**	66,74	13,67	60,59	16,58	78,02	15,00	82,55	8,36
	**Vesselness**	2,52	6,68	30,00	39,73	1,50	4,07	50,52	1,78
**K-net**	**Original OCTA**	54,13	14,07	41,13	14,61	86,21	10,90	76,70	6,66
	**Norm**	57,51	14,15	45,17	15,41	85,84	12,87	78,93	7,05
	**Proj art**	55,34	14,35	47,03	17,29	74,62	15,89	75,79	8,26
	**Contrast**	62,73	13,86	53,30	15,38	80,30	13,83	80,35	8,49
	**Vesselness**	14,43	14,00	32,93	23,06	29,49	38,42	55,25	6,97
**Swin**	**Original OCTA**	65,38	14,45	56,10	17,76	84,13	14,85	83,88	8,34
	**Norm**	66,73	14,75	58,75	18,00	82,07	15,21	83,77	8,68
	**Proj art**	69,06	14,70	63,00	18,08	80,36	15,82	84,38	8,84
	**Contrast**	68,15	15,11	61,00	18,56	82,36	16,02	84,46	8,88
	**Vesselness**	46,17	14,96	31,38	12,82	97,89	7,21	69,35	8,45
**Unet**	**Original OCTA**	56,70	17,04	42,40	16,93	94,68	12,51	80,05	8,28
	**Norm**	59,34	16,55	45,37	16,90	93,64	12,43	82,09	7,93
	**Proj art**	66,23	16,25	55,86	19,45	87,92	14,85	84,72	8,89
	**Contrast**	**70,05**	**15,30**	**63,64**	**17,35**	81,66	17,28	**84,97**	**9,92**
	**Vesselness**	22,18	10,53	31,32	13,79	22,44	15,72	54,18	5,32
**Unet 3D**	**Original OCTA**	56,42	15,08	46,39	18,64	82,48	18,17	85,36	8,92
	**Norm**	55,80	15,37	43,75	19,23	90,43	17,64	87,42	8,11
	**Proj art**	48,99	15,32	38,12	19,35	86,34	17,47	82,31	9,37
	**Contrast**	53,59	17,00	54,39	20,48	63,35	26,29	78,16	13,14
	**Vesselness**	38,05	17,61	44,09	15,56	42,94	30,10	68,79	13,85

Table S4 Performance of the 2D and 3D Unet model tested on the test set from different pathologies. Vascular density (VD), vasculature fragmentation (VF) and fractal dimension (FD) are reported.

**Net**	**Dataset**	**VD**		**VF**		**FD**	
		**mean**	**std**	**mean**	**std**	**mean**	**std**
**Convexnet**	**Original OCTA**	80,82	85,43	-40,80	32,30	4,86	5,51
	**Norm**	59,00	74,16	-36,84	33,98	3,14	5,26
	**Proj art**	54,24	65,00	-34,02	36,38	2,99	4,89
	**Contrast**	49,17	56,25	-21,85	39,65	3,21	4,37
	**Vesselnes**	350,86	243,20	-87,80	16,48	15,10	7,09
**Deeplabv3**	**Original OCTA**	157,38	138,96	-66,38	26,61	8,95	5,83
	**Norm**	106,36	90,21	-51,72	36,42	6,49	5,30
	**Proj art**	33,88	60,08	-23,22	38,98	**1,63**	**4,88**
	**Contrast**	39,75	49,42	-35,57	31,73	2,26	4,00

	**Vesselness**	-96,43	9,01	-99,16	1,61	-72,82	35,21
**K-net**	**Original OCTA**	145,75	132,96	-47,01	42,66	8,35	5,69
	**Norm**	119,08	106,55	-33,86	54,90	6,84	5,47
	**Proj art**	85,61	96,90	**-14,88**	**44,55**	4,87	6,15
	**Contrast**	66,47	65,32	-26,67	46,40	3,97	4,09
	**Vesselness**	80,21	312,00	-55,54	52,07	-24,97	39,47
**Swin**	**Original OCTA**	68,42	69,50	-35,65	35,71	4,16	4,75
	**Norm**	55,71	63,81	-36,40	34,08	2,93	4,41
	**Proj art**	39,06	48,69	-32,54	37,74	2,00	3,90
	**Contrast**	50,89	67,14	-24,06	43,73	2,89	4,52
	**Vesselness**	281,33	206,82	-79,80	22,93	13,49	6,66
**Unet**	**Original OCTA**	169,46	144,34	-48,89	47,23	10,42	6,28
	**Norm**	143,73	121,63	-46,09	40,79	9,28	5,89
	**Proj art**	80,60	83,39	-35,47	48,55	5,73	5,05
	**Contrast**	38,95	50,95	-25,07	44,49	2,71	4,02
	**Vesselness**	**-5,84**	**110,30**	180,79	241,26	-5,64	11,05
**Unet 3D**	**Original OCTA**	137,61	120,41	111,84	71,13	7,13	6,56
	**Norm**	175,61	127,63	120,74	61,55	9,17	6,63
	**Proj art**	223,70	165,12	39,67	64,03	10,39	7,66
	**Contrast**	50,82	78,90	339,33	416,19	0,97	7,91
	**Vesselness**	14,34	74,73	123,44	92,72	-0,93	6,61

Table S5 Performance of the 2D and 3D Unet model tested on the test set from different pathologies. CAL metrics are reported.

**Net**	**Dataset**	**CAL area**		**CAL conn**		**CAL length**		**CAL**	
		**mean**	**std**	**mean**	**std**	**mean**	**std**	**mean**	**std**
**Convexnet**	**Original OCTA**	69,56	17,15	99,80	0,15	60,46	14,00	43,85	15,79
	**Norm**	72,18	17,29	99,81	0,14	61,06	14,57	45,98	16,25
	**Proj art**	73,55	16,85	99,82	0,13	62,94	14,86	48,19	16,23
	**Contrast**	75,66	17,08	99,85	0,12	66,93	15,15	52,89	17,68
	**Vesselness**	41,97	15,30	99,64	0,21	33,53	12,43	15,19	9,27
**Deeplabv3**	**Original OCTA**	60,27	18,05	99,72	0,18	53,17	13,43	33,74	14,89
	**Norm**	65,23	17,14	99,77	0,15	55,96	13,42	38,12	15,10
	**Proj art**	69,90	15,61	99,84	0,12	59,12	13,54	43,03	14,45
	**Contrast**	74,42	15,65	99,83	0,13	62,49	14,11	48,29	15,54
	**Vesselness**	1,95	4,90	99,60	0,23	0,50	0,94	0,05	0,16
**K-net**	**Original OCTA**	57,83	15,91	99,77	0,15	49,08	10,99	29,62	11,62
	**Norm**	62,22	16,20	99,80	0,14	50,74	12,67	32,95	12,85
	**Proj art**	58,96	16,00	**99,86**	**0,12**	47,47	12,05	29,47	11,85

	**Contrast**	69,27	15,84	99,84	0,11	58,64	15,24	42,52	15,28
	**Vesselness**	14,49	14,04	99,72	0,22	11,24	11,38	3,10	3,90
**Swin**	**Original OCTA**	72,39	16,62	99,82	0,14	62,62	14,56	47,18	16,36
	**Norm**	73,40	17,20	99,82	0,13	59,81	15,95	45,83	17,09
	**Proj art**	**76,40**	**16,96**	99,83	0,13	65,21	15,28	52,03	17,23
	**Contrast**	75,08	17,54	99,84	0,12	62,74	15,85	49,19	17,91
	**Vesselness**	47,98	16,94	99,67	0,20	41,42	15,48	21,65	13,61
**Unet**	**Original OCTA**	59,74	18,86	99,76	0,17	55,22	16,20	35,68	18,40
	**Norm**	63,06	18,31	99,78	0,16	58,28	15,68	39,31	18,30
	**Proj art**	70,85	18,06	99,81	0,15	64,36	16,05	48,18	19,89
	**Contrast**	76,28	17,23	99,84	0,13	**67,98**	**16,13**	**54,41**	**19,48**
	**Vesselness**	23,98	11,84	99,35	0,85	20,82	10,86	6,15	6,10
**Unet 3D**	**Original OCTA**	57,94	17,61	99,997	0,003	28,32	9,21	17,83	9,83
	**Norm**	56,64	18,18	99,996	0,004	27,28	9,61	16,96	10,38
	**Proj art**	48,81	18,36	99,998	0,002	22,85	10,97	12,96	10,51
	**Contrast**	55,63	20,26	99,994	0,003	28,28	12,40	18,01	12,76
	**Vesselness**	38,29	20,59	99,997	0,002	16,29	11,34	8,39	8,45

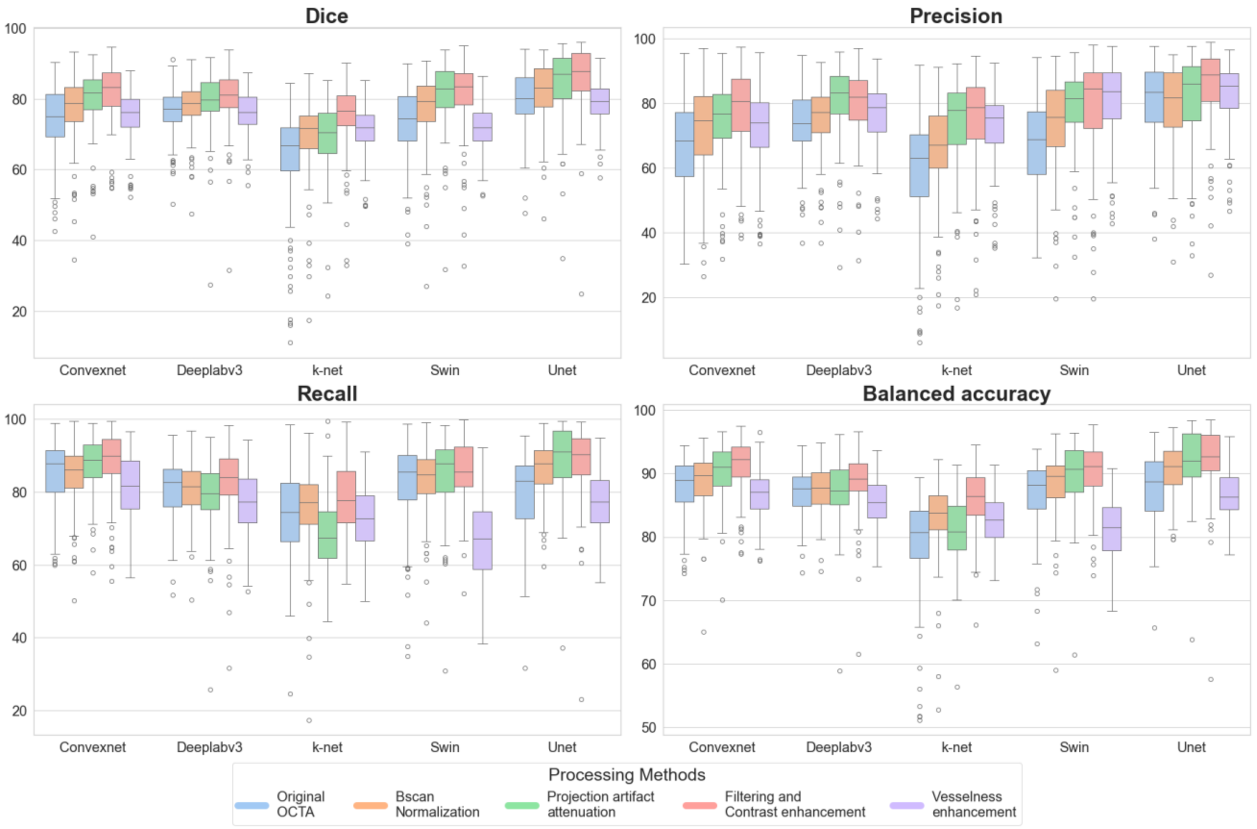

Fig. S2 Quantitative comparison of segmentation performance across different 2D approaches and preprocessing methods. Dice, precision, recall and balanced accuracy are reported. Different colors indicate different preprocessing methods, as shown in the legend.

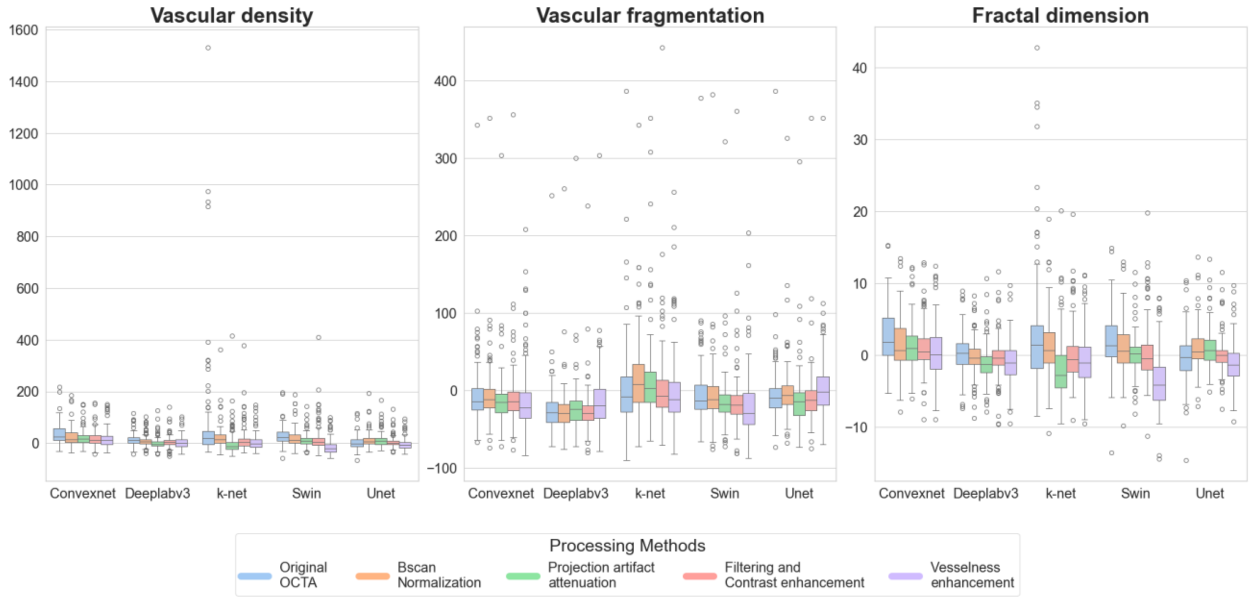

Fig. S3 Quantitative comparison of segmentation performance across different 2D approaches and preprocessing methods. Vascular density, vasculature fragmentation and fractal dimension are reported. Different colors indicate different preprocessing methods, as shown in the legend.

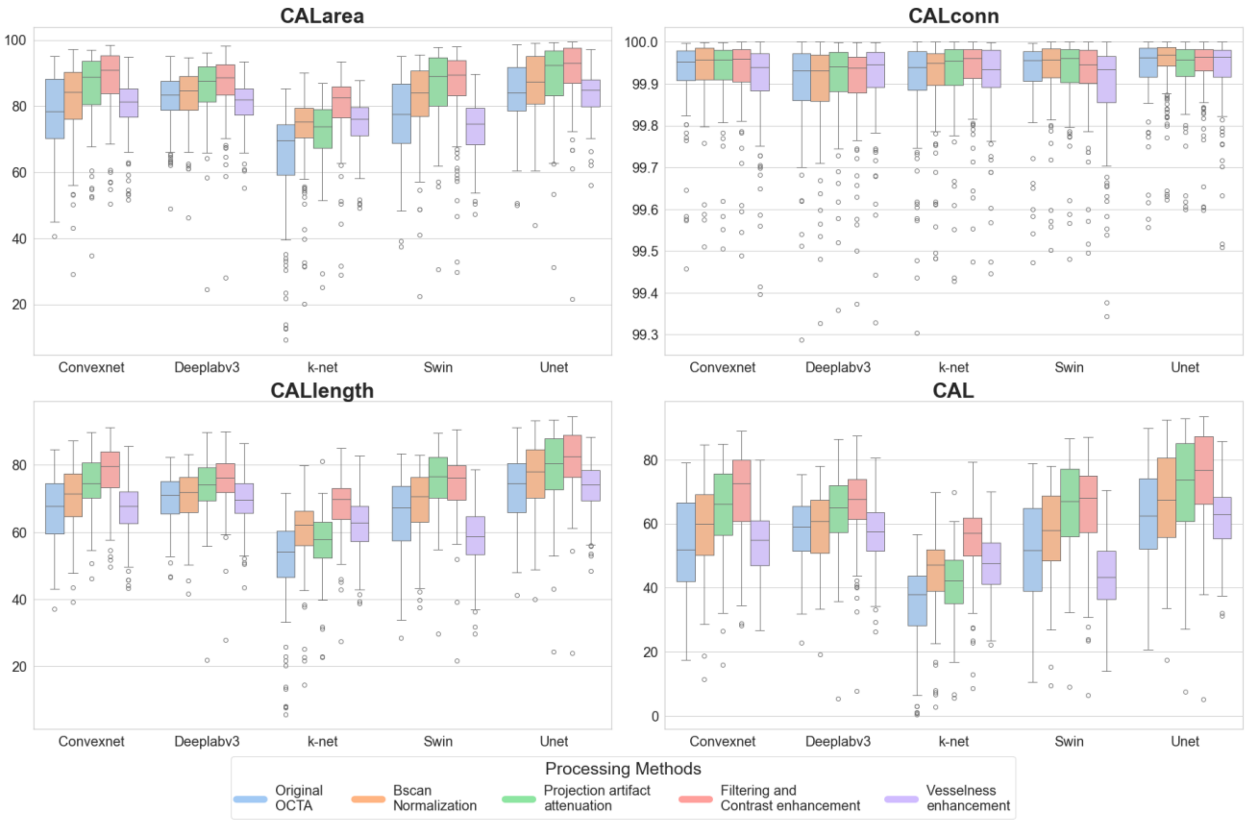

Fig. S4 Quantitative comparison of segmentation performance across different 2D approaches and preprocessing methods. CAL metrics are reported. Different colors indicate different preprocessing methods, as shown in the legend.

4. Comparison of evaluation metrics varying dataset size used for training

To evaluate the influence of the dataset set for the segmentation performance, the training set size was reduced to include 25%, 50% and 75% of the original training set, while leaving the validation and test set the same as before. With this division, the considered datasets were as shown in Table S6.

Table S6 Acquired data volumes divided into train, validation, test set and test set from different pathologies.

# OCTA	*Healthy*	*CVD C1*	*CVD C2*	*CVD C3*	*CVD C4a*	*CVD C4b*	*CVD C4c*	*CVD C5*	*CVD C6*	*Tot*
Training set (original)	*53*	*7*	*34*	*37*	*26*	*5*	*14*	*2*	*20*	***198***
Training set (75% of the original)	*29*	*7*	*24*	*30*	*17*	*5*	*14*	*2*	*20*	***148***
Training set (50% of the original)	*14*	*7*	*11*	*14*	*12*	*5*	*14*	*2*	*20*	***99***
Training set (25% of the original)	*10*	*4*	*7*	*7*	*5*	*5*	*5*	*2*	*5*	***50***
Validation set	*12*	*4*	*9*	*13*	*7*	*4*	*10*	*2*	*7*	***68***
Test set	*11*	*1*	*3*	*6*	*7*	*5*	*5*	*3*	*4*	***45***

The segmentation models were then retrained using the considered training subsets and the Dice and CAL parameters were computed as evaluation metrics. The results are shown in the boxplots in Figure S5.

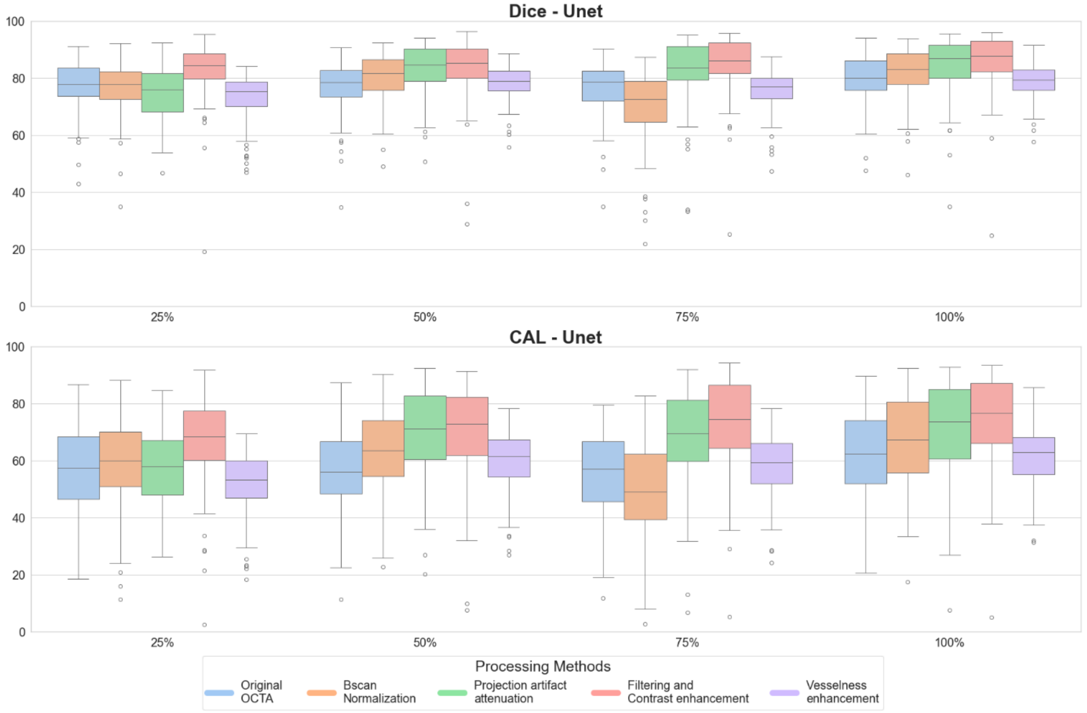

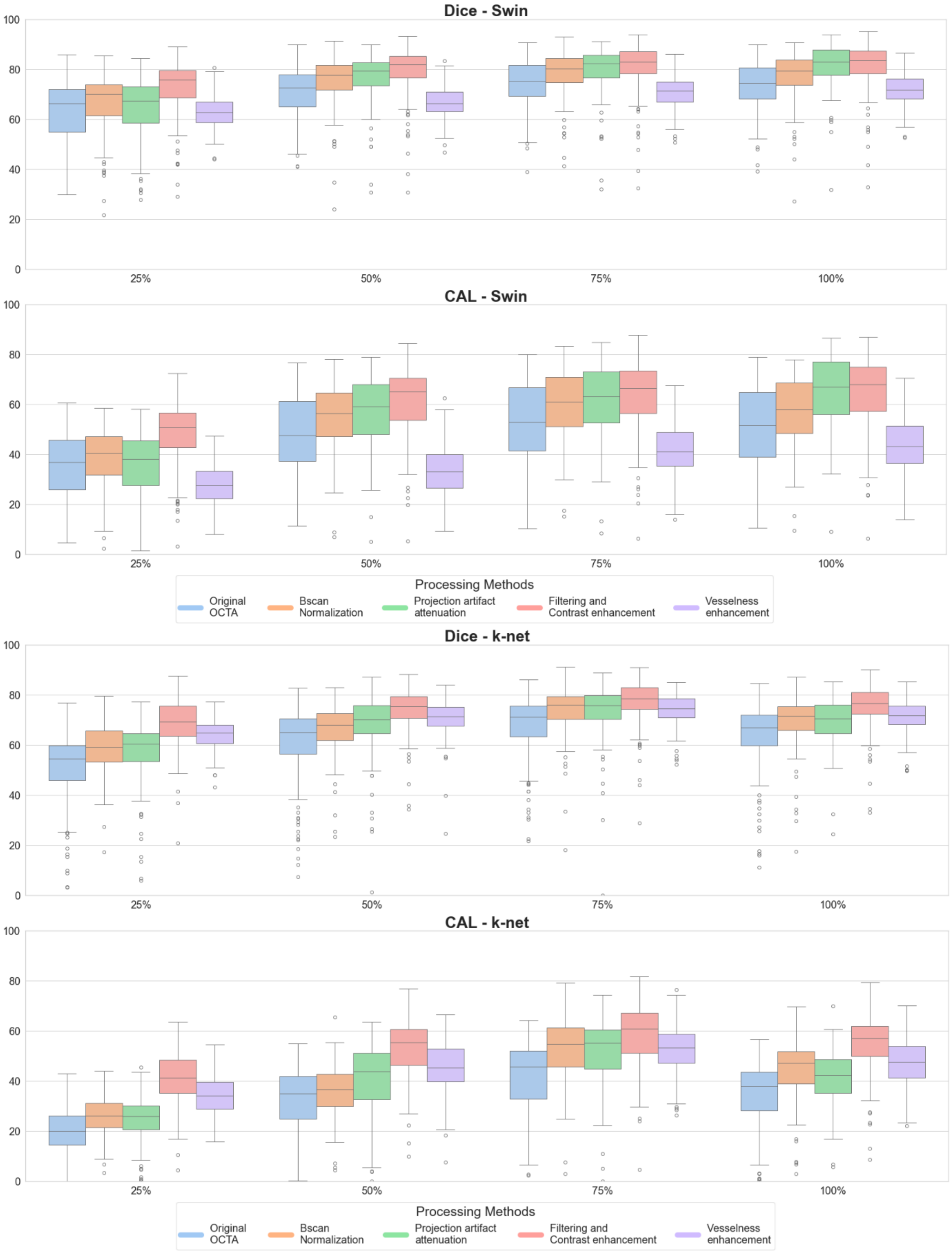
Dice - Swin
CAL - Swin
Dice - k-net
CAL - k-net
100
80
60
40
20
0
25%
50%
75%
100%
Processing Methods
Original OCTA
Bscan Normalization
Projection artifact attenuation
Filtering and Contrast enhancement
Vesselness enhancement

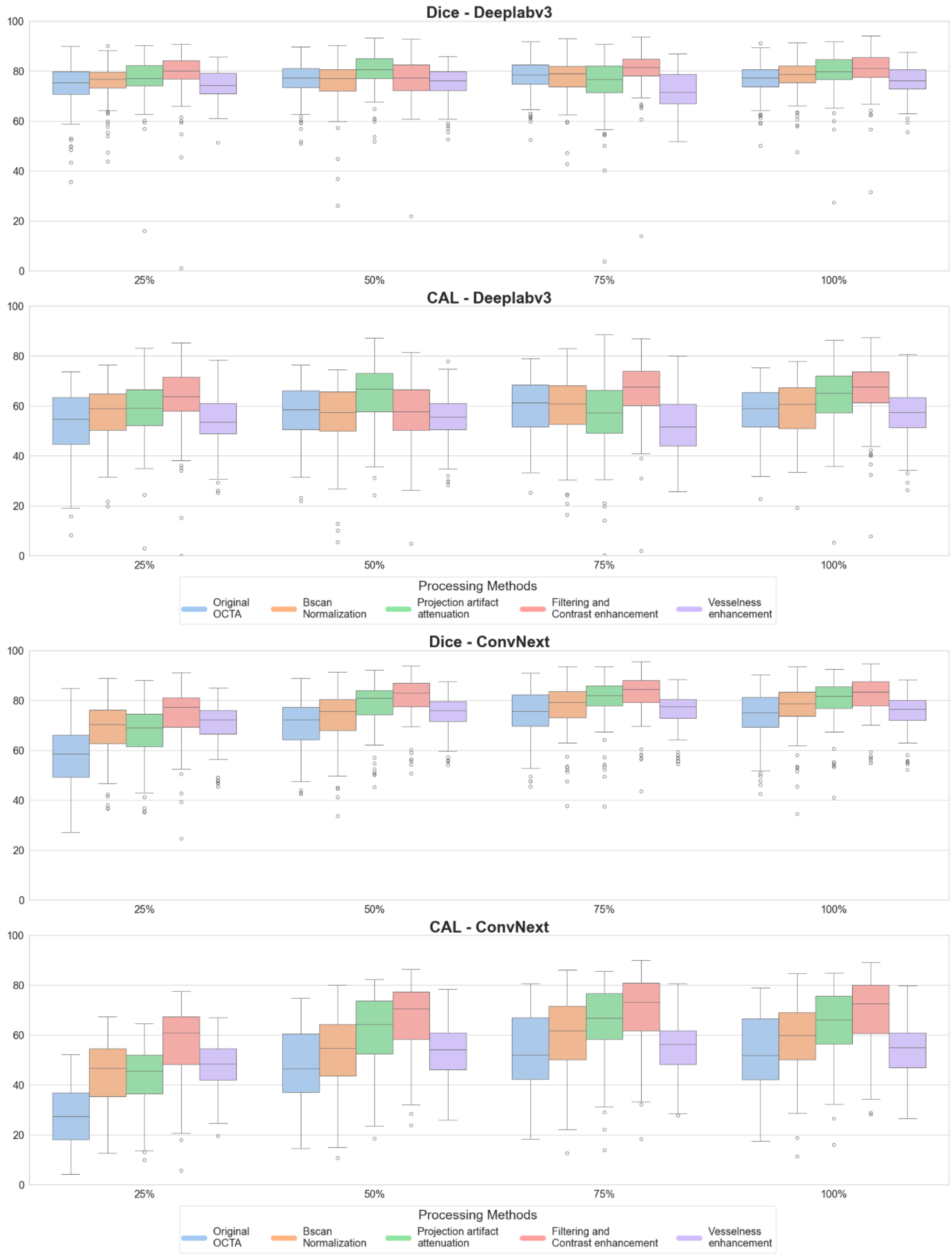

Fig. S5 Dice coefficient and CAL performance metrics comparison across different 2D approaches, preprocessing methods, and training set sizes (25%, 50%, 75%, 100% of the original training set). Different colors represent the preprocessing methods, as indicated in the legend.
